# Eating Disorder Risk and Its Biobehavioural Correlates in Italian University Students: The UniFoodWaste Study

**DOI:** 10.3390/nu18101588

**Published:** 2026-05-16

**Authors:** Flavia Pennisi, Antonio Pinto, Daniele Nucci, Lorenzo Stacchini, Marco Garzitto, Nicola Veronese, Stefania Maggi, Carlo Signorelli, Vincenzo Baldo, Marco Colizzi, Vincenza Gianfredi

**Affiliations:** 1 Faculty of Medicine, University Vita-Salute San Raffaele, 20132 Milan, Italy; pennisi.flavia@hsr.it (F.P.); pinto.antonio@hsr.it (A.P.); signorelli.carlo@hsr.it (C.S.); 2 PhD National Program in One Health Approaches to Infectious Diseases and Life Science Research Department of Public Health, Experimental and Forensic Medicine, University of Pavia, 27100 Pavia, Italy; daniele.nucci@ats-brescia.it; 3 Struttura Semplice Dipartimentale Igiene Alimenti e Nutrizione, Dipartimento di Igiene e Prevenzione Sanitaria, Agenzia di Tutela Della Salute (ATS) Brescia, Via Duca Degli Abruzzi, 15, 25124 Brescia, Italy; 4 Department of Community Healthcare Network, Health District “Toscana ASL Nord Ovest”, Pisa, Via Fleming 1, 56025 Pontedera, Italy; lorenzo.stacchini@uslnordovest.toscana.it; 5 Unit of Psychiatry, Department of Medicine (DMED), University of Udine, 33100 Udine, Italy; marco.garzitto@uniud.it; 6 Faculty of Medicine, Saint Camillus International University of Health Sciences, 00131 Rome, Italy; nicola.veronese@unicamillus.org; 7 Institute of Neuroscience, Aging Unit, National Research Council, 35127 Padova, Italy; stefania.maggi@in.cnr.it; 8 Department of Cardiac Thoracic Vascular Sciences and Public Health, University of Padua, 35128 Padua, Italy; vincenzo.baldo@unipd.it (V.B.); vincenza.gianfredi@unipd.it (V.G.); 9 Department of Psychosis Studies, Institute of Psychiatry, Psychology and Neuroscience, King’s College London, London SE5 8AF, UK

**Keywords:** eating disorders, university students, mental health, digital food environment, lifestyle factors, Italy

## Abstract

**Background/Objectives:** To assess the prevalence of eating disorder (ED) risk in a sample of Italian university students and to examine its independent associations with mental health indicators, self-rated health, body mass index (BMI), lifestyle behaviours, and engagement with digital food-related applications. **Methods:** Of the 2779 Italian university students who accessed the survey, 2691 completed and were included in the analysis. ED risk was assessed with the validated 5-item SCOFF questionnaire. Exposure included socio-demographics, BMI, depressive symptoms (PHQ-9), self-rated health, adherence to the Mediterranean diet (Medi-Lite), smoking, alcohol use (AUDIT-C), and use of food delivery and food waste apps. Multivariable logistic regression models, stratified by sex, and adjusted by age and education, estimated associations with ED risk. **Results:** Overall, 34.6% of participants screened positive for ED risk (women 39.5%, men 21.8%). Smoking and use of food delivery apps and food waste apps were independently associated with ED risk. Clinically relevant depressive symptoms (PHQ-9 ≥ 10) (aOR 3.37, 95% CI 2.82–4.02) and poor/fair self-rated health (aOR 2.45, 95% CI 1.93–3.11) showed the strongest association. Overweight (aOR 1.47, 95% CI 1.06–2.03) and obesity (aOR 2.48, 95% CI 1.53–4.01) increased the likelihood of ED risk. Risky alcohol use was also associated (aOR 1.42, 95% CI 1.15–1.75). **Conclusions:** More than one in three Italian university students is at risk for an ED, highlighting a substantial public health concern. Strong links with depression, perceived poor health, digital food app use, and unhealthy behaviours underscore the need for early screening and integrated mental health and nutrition interventions within university settings.

## 1. Introduction

Over recent decades, eating disorders (EDs) have shown a steady global increase [1], with an overall point prevalence estimated at 0.72% [2], and lifetime prevalence rates ranging from about 2% to 8% in women and from 0.7% to 2% in men [3].

These disorders are now among the leading causes of psychiatric morbidity in young people, exerting substantial effects on physical health, mental well-being, and quality of life [4,5]. In this age group, prevalence estimates rise sharply, with recent evidence reporting disordered eating in more than 20% of children and adolescents worldwide, and an increasing prevalence of eating disorders over recent decades [6].

University students represent a particularly vulnerable population [7,8]. The transition to adulthood is characterised by emerging autonomy in managing food and finances, the development of more stable social relationships, and academic challenges that may contribute to increased stress, alterations in dietary habits, and body dissatisfaction [9,10]. This is further compounded by a rapidly evolving social context, in which constant exposure to social media and idealised aesthetic standards may exacerbate the risk of dysfunctional behaviours [11,12].

The COVID-19 pandemic has further exacerbated this scenario, intensifying social isolation, economic uncertainty, and anxiety–depressive symptoms, factors that have been linked to an increase in episodes of uncontrolled eating and body image disturbances [13,14,15]. Such conditions are also associated with reduced attention to personal health and lower willingness to undergo preventive or diagnostic assessments [16,17].

Despite the relevance of the issue, data on the prevalence and correlates of EDs within the university population in Italy remain limited. Moreover, existing studies have rarely considered eating disorder risk within a broader framework that includes mental health, self-perceived health, lifestyle behaviours, adherence to the Mediterranean diet, and emerging features of the digital food environment, such as food delivery and food waste applications. Addressing this gap is relevant to inform prevention and early identification strategies in academic settings [18,19,20].

Therefore, the present study aimed to assess the prevalence of eating disorder risk in a large sample of Italian university students and to examine its association with sociodemographic characteristics, mental health indicators, self-rated health, body mass index, lifestyle behaviours, Mediterranean diet adherence, and the use of food-related digital applications. It employs validated screening tools for EDs and mental health, and examines both dietary habits and lifestyle factors, as well as innovative aspects such as the use of food-related applications.

## 2. Materials and Methods

### 2.1. Study Design and Data Collection

The UniFoodWaste study was a cross-sectional survey of Italian university students aged ≥ 18 years, enrolled in 2021/2022. Data were collected via an anonymous online questionnaire (Microsoft Forms, Microsoft Corporation, One Microsoft Way, Redmond, WA, USA) (between August and October 2023), distributed through institutional mailing lists, student associations, and academic networks, with access restricted to institutional accounts. Participation was voluntary with electronic informed consent. To limit missing data, all questions were mandatory. A detailed study protocol is published elsewhere [21].

### 2.2. Eating Disorder Risk

Participants completed the Italian version of the SCOFF questionnaire, a validated 5-item screening tool for ED risk with dichotomous answers (“Yes” = 1, “No” = 0; total score 0–5) [22,23]. Items cover feeling sick from overeating (Sick), loss of control (Control), recent weight loss ≥ 6 kg in 3 months (One stone), perceiving oneself as fat (Fat), and food preoccupation (Food). Higher scores indicate more ED symptoms. The Italian version reported a Cronbach’s α of 0.64, indicating moderate internal consistency [19]. Following validation studies, a cut-off ≥3 was used, with sensitivity 97.0% and specificity 87.5% for identifying ED risk [22,23,24].

### 2.3. Sociodemographic Variables

A comprehensive set of covariates was collected to account for potential sociodemographic, lifestyle, and health-related confounders. Sociodemographic information included gender (female, male, prefer not to say), age (18–23, 24–29, ≥30 years), geographical area of residence (north, central–south, abroad, according to ISTAT macro-areas), educational level (high school diploma, bachelor’s degree, master’s degree or higher), student status (resident in Milan, off-site, commuter, Erasmus), and living arrangement (alone, with family, with partner, or with friends/roommates).

### 2.4. Health-Related Variables

Health indicators included self-rated health (SRH), assessed with a single item (“In general, how would you rate your health?”; excellent to poor) [25,26], dichotomized into high (excellent/very good/good) and low (fair/poor). SRH is a validated predictor of health outcomes [27,28]. Body mass index (BMI) was calculated from self-reported weight and height (kg/m^2^) and classified per WHO standards: underweight (<18.5), normal (18.5–24.9), overweight (25.0–29.9), and obesity (≥30). Depressive symptoms were measured with the Italian Patient Health Questionnaire-9 (PHQ-9; score 0–27), with ≥10 indicating clinically relevant symptoms [29,30]. The PHQ-9 has shown good internal consistency in previous validation studies, with Cronbach’s α values ranging from 0.86 to 0.89 [29,30].

### 2.5. Lifestyle Variables

Lifestyle factors included smoking status (never, former, occasional, regular), alcohol use assessed with AUDIT-C (score 0–12; hazardous drinking ≥5 for men, ≥4 for women) [29], and use of food-related apps (delivery, waste; yes/no). Dietary patterns were assessed with the 9-item Medi-Lite questionnaire [31], yielding scores 0–18; ≥12 indicated high adherence to the Mediterranean diet, consistent with prior studies [32,33].

### 2.6. Statistical Analysis

Analyses used a complete case approach, excluding participants with missing data or lacking consent. Descriptive statistics summarised population characteristics: categorical variables as frequencies and percentages, continuous as means and SD, with normality tested via Kolmogorov–Smirnov. Group differences between students at risk of EDs (SCOFF ≥ 3) and not at risk (SCOFF < 3) were assessed with chi-square or Fisher’s exact tests for categorical variables, and t tests or one-way ANOVA for continuous variables. Bivariate analyses examined associations between ED risk and sociodemographics, BMI, self-rated health, depressive symptoms (PHQ-9), Mediterranean diet adherence (Medi-Lite), alcohol use (AUDIT-C), smoking, food app use, and geographical area. Chi-square with post hoc tests were used for categorical variables, Pearson’s correlations for continuous variables, with Bonferroni correction for multiple testing. Multivariable logistic regression assessed independent associations with ED risk, adjusting for age and education and stratified by sex; results are reported as adjusted odds ratios (aORs) with 95% CIs. Analyses were conducted in Stata version 18.5 (StataCorp LLC, 4905 Lakeway Drive, College Station, TX, USA), with two-sided *p* < 0.05 considered significant.

### 2.7. Sample Size

Sample size was calculated with a 95% confidence level and 5% margin of error, using the 60,988 students enrolled at the University of Milan in 2021/2022 as the target population [34]. A conservative estimate of 50% was used for the expected proportion, as this value maximises the required sample size and ensures adequate statistical power. Accordingly, the minimum required sample was 382 participants.

### 2.8. Ethical Considerations

The study was approved by the Ethics Committee of the University of Milan (Approval ID: 71.23; 20 June 2023), confirming compliance with established ethical standards and the protection of participants’ rights throughout the study process. Participation was voluntary, and all responses were anonymous, in accordance with the Declaration of Helsinki.

## 3. Results

### 3.1. Sample Characteristics

**Figure 1:**
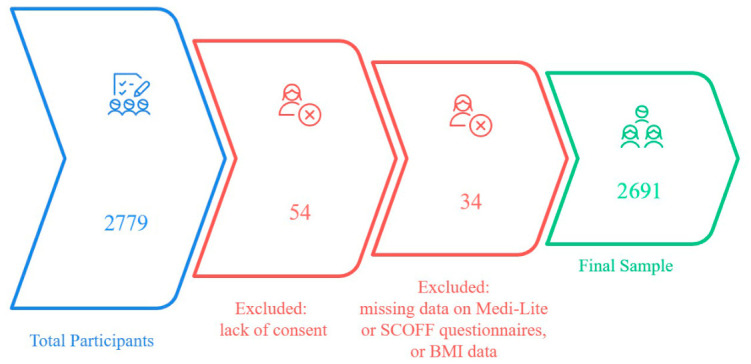
Flowchart of participant selection.

After excluding 54 participants without consent and 34 with missing data, 2691 students were analysed (Figure 1). Overall, 34.6% (n = 932) screened at high risk for EDs (SCOFF ≥ 3) and 65.4% (n = 1759) were not (SCOFF < 3). Prevalence was higher in women than men (39.5% vs. 21.8%, *p* < 0.001), in students aged 18–23 versus ≥30 years, and in those from central–southern versus northern Italy. High-risk students were more often smokers and users of food delivery and food waste apps. They also had higher BMI and PHQ-9 scores (9.9 vs. 6.3, *p* < 0.001); 44.4% reported clinically relevant depressive symptoms versus 18.9% in the low-risk group (*p* < 0.001). Poorer self-rated health was more common among high-risk students (33.8% vs. 45.4% very good/excellent, *p* < 0.001), as was risky alcohol use (19.3% vs. 14.6%, *p* = 0.001). Results are shown in Table 1.

### 3.2. SCOFF Item Analysis

**Figure 2:**
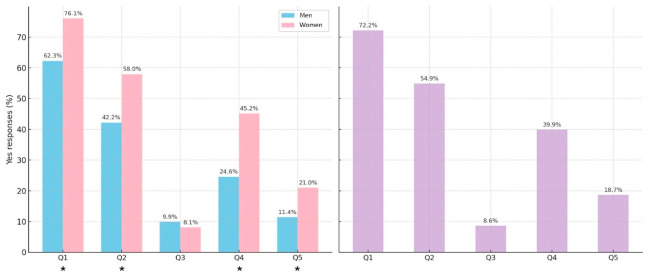
Distribution of positive responses to SCOFF items by gender and total. Statistically significant differences are reported using * (pink: women, blue: men; purple: total).

The distribution of positive responses to individual SCOFF items differed by sex (Figure 2). Women more frequently endorsed Q1, Q2, Q4 and Q5, while no significant difference was observed for Q3.

**Figure 3:**
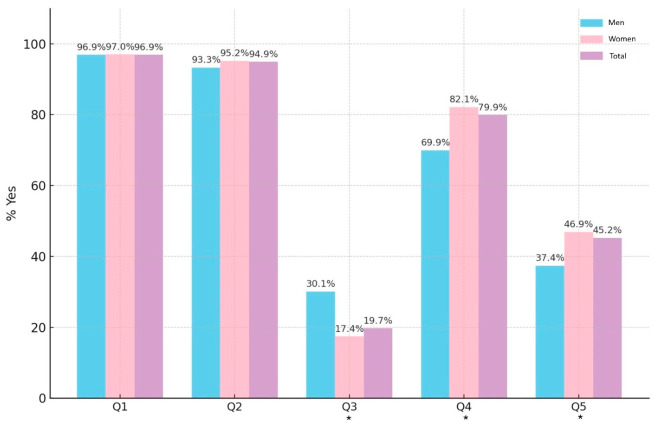
Distribution of “yes” responses to SCOFF items by gender (restricted to SCOFF ≥ 3). Statistically significant differences are reported using *.

Among students with SCOFF ≥ 3, nearly all endorsed the first two items, Q1 (96.9%) and Q2 (94.9%), with no significant gender differences (Figure 3). Men more frequently reported recent weight loss (Q3: 30.1% vs. 17.4%, *p* = 0.001), while women more often perceived themselves as fat despite being considered thin (Q4: 82.1% vs. 69.9%, *p* = 0.001) and reported that food dominated their life (Q5: 46.9% vs. 37.4%, *p* = 0.017).

### 3.3. SCOFF Combinations

**Figure 4:**
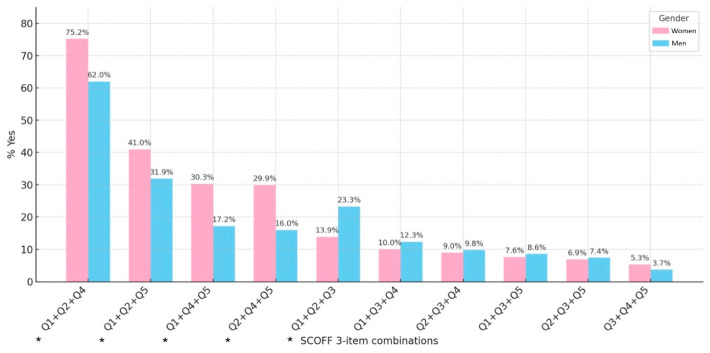
Most frequent 3-item SCOFF combinations among participants with SCOFF ≥ 3, stratified by gender. Statistically significant differences are reported using *.

The most frequent 3-item SCOFF combinations also differed by gender (Figure 4). Among women, the predominant pattern was Q1 + Q2 + Q4 (75.2%), whereas among men Q1 + Q2 + Q4 remained the most common but with lower prevalence (62.0%, *p* < 0.001). Other combinations including Q5 were significantly more frequent in women, while combinations involving Q3 (recent weight loss) were more frequent in men.

### 3.4. Multivariable Associations

Multivariable analyses showed that students from central–southern Italy had higher odds of ED risk than those from the north. Smoking was associated with increased risk in women and the total sample. Use of food delivery apps and food waste apps was also linked to higher risk. Depressive symptoms (PHQ-9 ≥ 10) showed the strongest associations (men: aOR = 2.38, 95% CI 1.62–3.50; women: aOR = 3.60, 95% CI 2.92–4.43; total: aOR = 3.37, 95% CI 2.82–4.02), as did fair/poor self-rated health (men: aOR = 2.77, 95% CI 1.69–4.55; women: aOR = 2.28, 95% CI 1.72–3.01; total: aOR = 2.45, 95% CI 1.93–3.11). BMI showed sex-specific effects: in women, overweight and obesity increased risk; in men, only obesity was significant. In the total sample, both overweight and obesity were associated. Risky alcohol use was also significant in women and the total sample. Among men, cohabitation was significantly associated with eating disorder risk in the bivariate analysis (*p* = 0.020); however, in the adjusted model, only living with family remained associated with lower odds of eating disorder risk compared with living alone (aOR = 0.50, 95% CI 0.29–0.86). Results are shown in Table 2.

## 4. Discussion

Evidence from a large sample of university students indicates that more than one in three screened positive on the SCOFF questionnaire, suggesting that a considerable proportion of young adults may be at risk of developing an ED. This prevalence is consistent with estimates reported in comparable university populations [35]. These findings further confirm that ED attitudes represent a significant public health concern, which in some cases may necessitate urgent care, a need that could have been exacerbated by the COVID-19 pandemic [36,37,38].

Consistent with previously consolidated evidence, female students showed a greater risk than their male counterparts [39,40]. However, the finding that over 20% of men screened positive underscores that ED patterns are not exclusive to women and may often go under-detected in male populations [41,42]. ED patterns were also more prevalent among younger students compared to older ones, confirming the heightened vulnerability to mental health problems [43,44]. Differences emerged when comparing students by region of provenance, with those from central and southern Italy being more likely to score above the cut-off for ED [45]. These findings may reflect cultural influences and differential exposure to social stressors [46], in line with recent indications that psychiatric diagnostic manuals may require revision [47].

Students’ lifestyles were associated with the risk of EDs, particularly among females. Specifically, smoking, food delivery app use, and food waste app use were each independently linked to higher odds of ED, possibly reflecting unhealthy behaviours or maladaptive coping strategies [48,49]. The association with food-related apps may reflect shifts in food environments and eating habits among younger generations [50,51]. Of interest, research on the unintended negative consequences of food-related apps suggests that their strong focus on eating behaviours may trigger or exacerbate ED and related psychological symptoms [52,53]. Nevertheless, the use of food delivery and food waste applications was assessed using dichotomous yes/no variables. Therefore, we were unable to examine the frequency, intensity, duration, motivations, or specific patterns of use. This limits the interpretation of the observed associations and prevents conclusions regarding the directionality or mechanisms linking app use and eating disorder risk. Moreover, the association between food waste application use and eating disorder risk should be interpreted with caution. Given the cross-sectional design, we cannot determine the direction of this association. While food-related applications may shape eating behaviours through increased exposure to food cues or greater monitoring of food choices, reverse causality is also plausible. Students with disordered eating symptoms or heightened preoccupation with food may be more likely to use food-related applications more frequently or more intensively [54]. Therefore, these findings should be considered hypothesis-generating and require confirmation in longitudinal studies.

A relevant habit associated with increased risk of ED was risky alcohol consumption, aligning with previous evidence for a role of substance misuse, possibly reflecting shared underpinnings such as impulsivity, maladaptive coping, or social influences [55,56]. The absence of this association in men may reflect gender differences in drinking motives and their relationship to ED [57].

A key finding of this study is the strong link between disordered eating and health difficulties, with students at risk of depression showing a threefold higher likelihood, and those perceiving poor health also being more vulnerable [58,59]. These results support a bidirectional relationship between EDs, poor mental health, and heightened illness perception [60]. Notably, the clustering of depressive symptoms and ED in university students has been highlighted in a large network analytic study employing methodology similar to the present study [61], reinforcing the interconnectedness of these conditions.

In terms of ED-related core symptoms, we found a not unequivocal association between BMI and SCOFF scores, consistent with clinical evidence. However, while mean BMI did not differ in univariate analyses, categorical analyses showed that overweight and obesity increased ED risk, in line with previous findings [62] and confirmed in multivariable models. Moreover, sex-specific patterns emerged: in female students, BMI was linked to risk both continuously and categorically, while in men only obesity was significant, confirming and extending previous evidence of gender-driven differences in how increased BMI relates to ED symptoms [63,64]. However, BMI was calculated from self-reported weight and height, which may be affected by systematic reporting bias. This limitation is particularly relevant in the context of eating disorder research, as individuals with body image concerns or disordered eating symptoms may underreport weight and/or misreport height [65,66]. Consequently, BMI categories may have been misclassified, potentially leading to an underestimation or distortion of the associations between BMI and eating disorder risk. SCOFF risk was not unequivocally associated with adherence to the Mediterranean diet (Medi-Lite score), suggesting that psychological and behavioural factors may be more relevant than diet quality [67,68]. However, this finding should be interpreted with caution. The Medi-Lite questionnaire measures adherence to the Mediterranean dietary pattern but does not assess key features of disordered eating, such as caloric restriction, binge eating, compulsive or rigid eating behaviours, food-related distress, or compensatory practices. Therefore, the absence of an association between Medi-Lite score and SCOFF risk does not necessarily indicate that diet quality or eating behaviours are unrelated to eating disorder risk. This finding warrants further investigation, as a review on the association between the Mediterranean diet and major psychiatric disorders [69] identified only one study on EDs, which suggested a protective effect [70]. In addition, the SCOFF questionnaire is a brief screening instrument and does not provide a clinical diagnosis of eating disorders. The Italian version has reported moderate internal consistency (Cronbach’s α = 0.64), below the commonly used threshold of 0.70. This may have introduced measurement error and could have affected the classification of eating disorder risk. Therefore, prevalence estimates and associations based on the SCOFF should be interpreted as indicators of screening positivity rather than confirmed eating disorder diagnoses.

Analysis of SCOFF items showed gender-specific patterns, with women more often endorsing body image distortion and food preoccupation, while men reported recent weight loss. The most frequent combination (Q1 + Q2 + Q4) indicated a core cluster of loss of control, compensatory behaviours, and body image distortion, in line with the previous literature highlighting these as central features of ED [71,72].

Key strengths include the large sample, validated screening tools, and a student population drawn from across Italy, enhancing national representativeness. However, some limitations should be acknowledged. The cross-sectional design limits causal inference, and self-reports may be affected by recall and social desirability bias [73,74]. As a screening tool, the SCOFF does not provide a clinical diagnosis, so prevalence estimates should be interpreted with caution. Due to the survey dissemination strategy (i.e., distribution through institutional mailing lists, student associations, and academic networks), it was not possible to calculate a formal response rate or to assess non-response patterns. This prevents the evaluation of potential non-response bias and may limit the generalizability of the findings.

## 5. Conclusions

This study shows that a substantial proportion of students in this university-based sample screened positive for eating disorder risk. ED risk was associated with depressive symptoms, poorer self-rated health, smoking, risky alcohol use, higher BMI, and food-related application use, with some sex-specific differences. These findings support the need for early identification strategies in university settings and suggest that student health services may benefit from integrated approaches combining mental health support, nutritional counselling, and health promotion activities addressing lifestyle behaviours. Longitudinal studies are needed to clarify causal pathways and inform targeted preventive actions.

## Figures and Tables

**Table 1:** Descriptive characteristics of the sample, stratified by SCOFF.

	No Eating Disorders SCOFF < 3 N = 1759 (65.4)	Eating Disorders SCOFF ≥ 3 N = 932 (34.6)	*p*-Value
**Gender**			
Women	1148 (65.3)	751 (80.6)	
Men	585 (33.3)	163 (17.5)	<0.001
Prefer not to say	26 (1.5)	18 (1.9)	
**Age**			
18–23	889 (50.5)	523 (56.1)	
24–29	563 (32.0)	292 (31.3)	0.002
≥30	307 (17.5)	117 (12.6)	
**Geographical area**			
North	1644 (93.5)	836 (89.7)	0.002
Centre and south	99 (5.6)	86 (9.2)	
Abroad	16 (0.9)	10 (1.1)	
**Educational level**			
High school diploma	903 (51.3)	512 (54.9)	
Bachelor’s degree	451 (25.6)	240 (25.8)	0.068
Master’s degree or higher	405 (23.0)	180 (19.3)	
**Student status**			
Resident in Milan	461 (26.2)	236 (25.3)	
Off-site	368 (20.9)	223 (23.9)	0.298
Commuter	925 (52.6)	469 (50.3)	
Erasmus	5 (0.3)	4 (0.4)	
**Cohabitation**			
Alone	153 (8.7)	77 (8.3)	
Family	1211 (68.9)	630 (67.6)	0.121
Partner	220 (12.5)	105 (11.3)	
Friend(s) or roommate(s)	175 (10.0)	120 (12.9)	
**Smoking status**			
Not smoker	1307 (74.3)	615 (66.0)	<0.001
Smoker	452 (25.7)	317 (34.0)	
**Food delivery app**			
No	1053 (59.9)	490 (52.6)	<0.001
Yes	706 (40.1)	442 (47.4)	
**Food waste app**			
No	1150 (65.4)	556 (59.7)	0.003
Yes	609 (34.6)	376 (40.3)	
**Medi-Lite score** (mean and standard deviation)	10.3 (2.1)	10.2 (2.2)	0.181
**Medi-Lite**			
Low adherence	1252 (71.2)	686 (73.6)	0.182
High adherence	507 (28.8)	246 (26.4)	
**BMI** (mean and standard deviation)	21.8 (3.3)	22.4 (4.0)	<0.001
**BMI category**			
Underweight	240 (13.6)	99 (10.6)	
Normal weight	1266 (72.0)	662 (71.0)	0.001
Overweight	210 (11.9)	127 (13.6)	
Obese/Very obese	43 (2.4)	44 (4.7)	
**PHQ-9** (mean and standard deviation)	6.3 (4.4)	9.9 (5.6)	<0.001
**PHQ Score**			
PHQ-9 < 10	1426 (81.1)	518 (55.6)	<0.001
PHQ-9 ≥ 10	333 (18.9)	414 (44.4)	
**Self-rated health (SRH)**			
Poor	17 (1.0)	25 (2.7)	
Fair	187 (10.6)	167 (17.9)	
Good	756 (43.0)	425 (45.6)	<0.001
Very good	641 (36.4)	276 (29.6)	
Excellent	158 (9.0)	39 (4.2)	
**AUDIT-C**			
No risk	1503 (85.5)	752 (80.7)	0.001
At risk *	256 (14.6)	180 (19.3)	

* ≥5 for men or ≥4 for women.

**Table 2:** Associations between eating disorder risk (SCOFF ≥ 3) and sociodemographic, lifestyle, and health-related variables among Italian university students, stratified by sex and in the total sample. Results are reported as prevalence and adjusted odds ratios (aORs) with 95% confidence intervals. Odds ratios are adjusted for educational level and age. Statistical significant associations are reported in bold.

Men					Women				Total			
	SCOFF < 3 n = 585	SCOFF ≥ 3 n = 163	*p*-Value	AOR for SCOFF ≥ 3 and 95% CI	SCOFF < 3 n = 1148	SCOFF ≥ 3 n = 751	*p*-Value	AOR for SCOFF ≥ 3 and 95% CI	SCOFF < 3	SCOFF ≥ 3	*p*-Value	AOR for SCOFF ≥ 3 and 95% CI
Geographical area												
North	541 (79.0)	144 (21.0)		1	1077 (61.4)	676 (38.6)		1	1644 (66.3)	836 (33.7)		1
Centre and south	35 (67.3)	17 (32.7)		1.80 (0.98–3.33)	64 (48.9)	67 (51.2)		**1.71 (1.19–2.45)**	99 (53.5)	86 (46.5)		**1.76 (1.30–2.39)**
Abroad	9 (81.8)	2 (18.2)	0.139	0.87 (0.18–4.12)	7 (46.7)	8 (53.3)	0.010	1.98 (0.71–5.53)	16 (61.5)	10 (38.5)	0.002	1.39 (0.62–3.11)
**Student status**												
Resident in Milan	159 (78.7)	43 (21.3)		1	297 (61.1)	189 (38.9)		1	461 (66.1)	236 (34.0)		1
Off-site	120 (75.0)	40 (25.0)		1.21 (0.74–1.99)	244 (57.8)	178 (42.2)		1.09 (0.83–1.43)	368 (62.3)	223 (37.7)		1.14 (0.91–1.44)
Commuter	305 (79.4)	79 (20.6)	0.519	0.93 (0.74–1.99)	603 (61.3)	381 (38.7)	0.654	0.92 (0.73–1.16)	925 (66.4)	469 (33.6)	0.298	0.93 (0.76–1.13)
Erasmus	1 (50.0)	1 (50.0)		3.15 (0.19–52.46)	4 (57.1)	3 (42.9)		1.16 (0.25–5.29)	5 (55.6)	4 (44.4)		1.56 (0.41–5.90)
**Cohabitation**												
Alone	59 (70.2)	25 (29.8)		1	92 (64.3)	51 (35.7)		1	153 (66.5)	77 (33.5)		1
Family	409 (81.3)	94 (18.7)		0.50 (0.29–0.86)	784 (60.0)	523 (40.0)		1.03 (0.71–1.50)	1211 (65.8)	630 (34.2)	0.121	0.88 (0.65–1.20)
Partner	60 (76.0)	19 (24.1)	0.020	0.84 (0.41–1.73)	158 (65.1)	85 (35.0)	0.145	1.02 (0.66–1.57)	220 (67.7)	105 (32.3)		1.02 (0.71–1.47)
Friend(s) or roommate(s)	57 (69.5)	25 (30.5)		0.96 (0.49–1.88)	114 (55.3)	92 (44.7)		1.28 (0.82–2.00)	175 (59.3)	120 (40.7)		1.20 (0.83–1.74)
**Smoking status**												
Not smoker	409 (78.7)	111 (21.4)		1	882 (64.1)	495 (36.0)	<0.001	1	1307 (68.0)	615 (32.0)		1
Smoker	176 (77.2)	52 (22.8)	0.656	1.10 (0.76–1.60)	266 (51.0)	256 (49.0)		**1.68 (1.37–2.07)**	452 (58.8)	317 (41.2)	<0.001	**1.48 (1.24–1.76)**
**Food delivery app**												
No	353 (79.3)	92 (20.7)		1	683 (63.7)	389 (36.3)		1	1053 (68.2)	490 (31.8)		1
Yes	232 (76.6)	71 (23.4)	0.370	1.18 (0.83- 1.67)	465 (56.2)	362 (43.8)	0.001	**1.39 (1.15–1.68)**	706 (61.5)	442 (38.5)	<0.001	**1.37 (1.16–1.61)**
**Food waste app**												
No	412 (78.8)	111 (21.2)		1	722 (62.4)	436 (37.7)		1	1150 (67.4)	556 (32.6)	0.003	1
Yes	173 (76.9)	52 (23.1)	0.566	1.11 (0.76–1.63)	426 (57.5)	315 (42.5)	0.035	**1.22 (1.01–1.47)**	609 (61.8)	376 (38.2)		**1.28 (1.09–1.51)**
**Medi-Lite** (continuous)	10.13 (2.14) ^§^	9.82 (2.18) ^§^	0.106	0.93 (0.86–1.01)	10.41 (2.10) ^§^	10.29 (2.25) ^§^	0.261	0.98 (0.94–1.02)	10.32 (2.11) ^§^	10.20 (2.23) ^§^	0.1986	0.98 (0.94–1.03)
**Medi-Lite**												
Low adherence	436 (77.0)	130 (23.0)	0.142	1	800 (59.7)	541 (40.3)	0.299	0.90 (0.73–1.10)	1252 (64.6)	686 (35.4)		1
High adherence	152 (82.2)	33 (17.8)		0.72 (0.47–1.11)	349 (62.2)	212 (37.8)			507 (67.3)	246 (32.7)	0.182	0.92 (0.75–1.13)
**BMI** (continuous)	23.18 (8.63) ^§^	23.88 (3.38) ^§^	0.310	1.01 (0.99–1.03)	21.54 (4.65) ^§^	22.06 (4.33) ^§^	0.014	**1.04 (1.01–1.06)**	21.83 (3.31) ^§^	22.38 (3.97) ^§^	0.0002	**1.04 (1.02–1.07)**
**BMI category**												
Underweight	44 (88.0)	6 (12.0)		1	193 (67.7)	92 (32.3)		1	240 (70.8)	99 (29.2)		1
Normal weight	435 (80.4)	106 (19.6)		1.81 (0.75–4.39)	812 (59.9)	544 (40.1)		**1.42 (1.09–1.87)**	1266 (65.7)	662 (34.3)		1.27 (0.98–1.63)
Overweight	92 (69.2)	41 (30.9)	0.001	**3.57 (1.39–9.19)**	115 (58.4)	82 (41.6)	0.007	**1.62 (1.10–2.36)**	210 (62.3)	127 (37.7)	0.001	**1.47 (1.06–2.03)**
Obese/Very obese	14 (58.3)	10 (41.7)		**4.84 (1.49–15.75)**	28 (45.9)	33 (54.1)		**2.72 (1.55–4.79)**	43 (49.4)	44 (50.6)		**2.48 (1.53–4.01)**
**PHQ** **Score**												
PHQ-9 < 10	477 (82.0)	105 (18.0)	<0.001	1	934 (69.4)	411 (30.6)	<0.001	1	1426 (73.4)	518 (26.7)	<0.001	1
PHQ-9 ≥ 10	111 (65.7)	58 (34.3)		**2.38 (1.62–3.50)**	215 (38.6)	342 (61.4)		**3.60 (2.92–4.43)**	333 (44.6)	414 (55.4)		**3.37 (2.82–4.02)**
**SRH**												
Excellent/Very good	278 (82.3)	60 (17.8)		1	510 (66.9)	252 (33.1)		1	799 (71.7)	315 (28.3)		1
Good	240(78.8)	65 (21.3)		1.27 (0.86–1.88)	503 (60.0)	350 (41.0)		**1.41 (1.15–1.73)**	756 (64.0)	425 (36.0)		**1.43 (1.20–1.71)**
Fair/Poor	67 (63.8)	38 (36.2)	<0.001	**2.77 (1.69–4.55)**	135 (47.5)	149 (52.5)	<0.001	**2.28 (1.72–3.01)**	204 (51.5)	192 (48.5)	<0.001	**2.45 (1.93–3.11)**
**AUDIT-C**												
No risk	484 (77.7)	139 (22.3)	0.442	1	993 (62.5)	595 (37.5)	<0.001	1	1503 (66.7)	752 (33.4)	0.001	1
At risk *	101 (80.8)	24 (19.2)		0.82 (0.51–1.33)	155 (49.8)	156 (50.2)		**1.67 (1.31–2.14)**	256 (58.7)	180 (41.3)		**1.42 (1.15–1.75)**

* ≥5 for men or ≥4 for women ^§^ mean and standard deviation.

## Data Availability

The data supporting the findings of this study are not publicly available due to ethical and privacy restrictions. The dataset contains sensitive information collected from human participants and cannot be shared openly to protect individual confidentiality. De-identified data may be made available by the corresponding author upon reasonable request and pending approval from the institutional ethics committee.

## Abbreviations

The following abbreviations are used in this manuscript:

ED	Eating disorder
EDs	Eating disorders
BMI	Body mass index
PHQ-9	Patient Health Questionnaire-9
Medi-Lite	Mediterranean Diet Adherence Score
AUDIT-C	Alcohol Use Disorders Identification Test-Consumption
SRH	Self-rated health
SCOFF	Sick, Control, One stone, Fat, Food questionnaire
SD	Standard deviation
ANOVA	Analysis of variance
aOR	Adjusted odds ratio
CI	Confidence interval
WHO	World Health Organization
COVID-19	Coronavirus disease 2019
ATS	Agenzia di Tutela della Salute
DMED	Department of Medicine
